# A Survey on the Role of Industrial IoT in Manufacturing for Implementation of Smart Industry

**DOI:** 10.3390/s23218958

**Published:** 2023-11-03

**Authors:** Muhammad Shoaib Farooq, Muhammad Abdullah, Shamyla Riaz, Atif Alvi, Furqan Rustam, Miguel Angel López Flores, Juan Castanedo Galán, Md Abdus Samad, Imran Ashraf

**Affiliations:** 1 Department of Computer Science, University of Management and Technology, Lahore 54000, Pakistan; shoaib.farooq@umt.edu.pk (M.S.F.); abdullah.muhammad001144@gmail.com (M.A.); shamyla.riaz@umt.edu.pk (S.R.); atif.alvi@umt.edu.pk (A.A.); 2 School of Computer Science, University College Dublin, D04 V1W8 Dublin, Ireland; furqan.rustam1@gmail.com; 3 Research Group on Foods, Universidad Europea del Atlantico, Isabel Torres 21, 39011 Santander, Spain; miguelangel.lopez@uneatlantico.es (M.A.L.F.); juan.castanedo@uneatlantico.es (J.C.G.); 4 Research Group on Foods, Universidad Internacional Iberoamericana, Campeche 24560, Mexico; 5 Instituto Politécnico Nacional, UPIICSA, Ciudad de México 04510, Mexico; 6 Universidad Internacional Iberoamericana, Arecibo, PR 00613, USA; 7 Department of Projects, Universidade Internacional do Cuanza, Cuito EN250, Bie, Angola; 8 Department of Information and Communication Engineering, Yeungnam University, Gyeongsan 38541, Republic of Korea

**Keywords:** Internet of Things, industrial IoT, smart industry, network protocols

## Abstract

The Internet of Things (IoT) is an innovative technology that presents effective and attractive solutions to revolutionize various domains. Numerous solutions based on the IoT have been designed to automate industries, manufacturing units, and production houses to mitigate human involvement in hazardous operations. Owing to the large number of publications in the IoT paradigm, in particular those focusing on industrial IoT (IIoT), a comprehensive survey is significantly important to provide insights into recent developments. This survey presents the workings of the IoT-based smart industry and its major components and proposes the state-of-the-art network infrastructure, including structured layers of IIoT architecture, IIoT network topologies, protocols, and devices. Furthermore, the relationship between IoT-based industries and key technologies is analyzed, including big data storage, cloud computing, and data analytics. A detailed discussion of IIoT-based application domains, smartphone application solutions, and sensor- and device-based IIoT applications developed for the management of the smart industry is also presented. Consequently, IIoT-based security attacks and their relevant countermeasures are highlighted. By analyzing the essential components, their security risks, and available solutions, future research directions regarding the implementation of IIoT are outlined. Finally, a comprehensive discussion of open research challenges and issues related to the smart industry is also presented.

## 1. Introduction

The Internet of Things (IoT), originally introduced in the early 1990s, acquired significant attention during the late 1990s after an investigation by the Massachusetts Institute of Technology (MIT), Auto-ID Labs, which raised its overall publication market [1]. Conceptually, the IoT is a combination of virtual domains that use the internet to exchange information. Various real-world applications have adopted IoT-based technologies that have made life easy. The wide applications of the IoT include smart healthcare, smart agriculture, automatic security systems, smart factories, and smart industries [2].

Although a lot of work has been conducted in the IoT-enabled smart industry, further efforts are needed to overcome issues related to security and privacy [3]. The smart industry has initiated an extremely positive effort by integrating IoT technology in the industrial domain. As predicted, advanced technologies and industry could solve numerous problems by implementing pervasive security countermeasures through the effective implementation of the IoT [4]. The state-of-the-art implementation of the IoT is solving industrial security issues by providing productive and cost-effective solutions [5]. The industrial IoT process depends on the cyber-physical system (CPS). Therefore, the CPS is considered a pillar of IIoT and is used in the industrial wireless network to monitor and control the physical processes among IoT devices, sensors, controllers, and actuators [6]. Furthermore, IIoT provides cost-effective and well-organized scheduling of limited resources to boost production.

**Figure 1:**
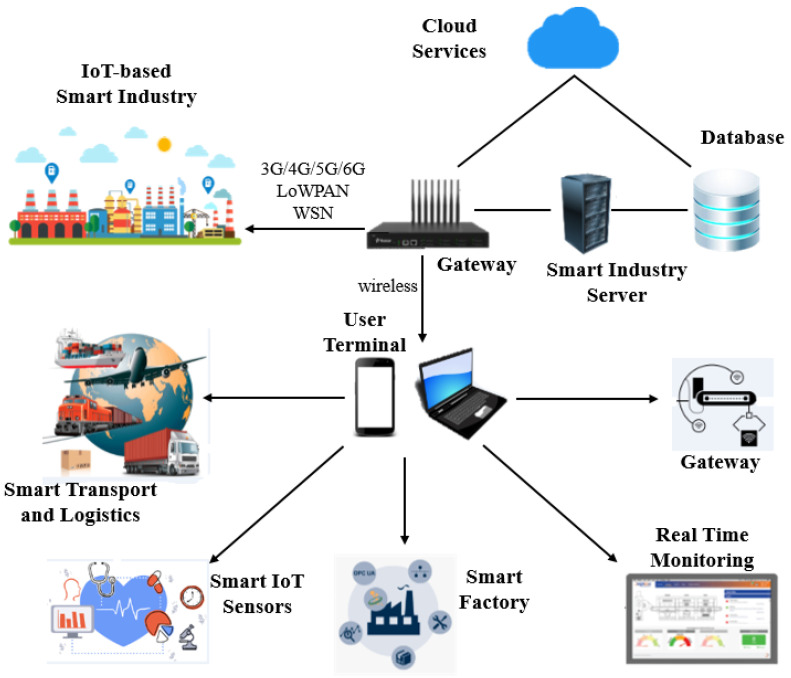
Industrial IoT trends show recent applications for industrial IoT.

Figure 1 shows the recent IIoT trends, which offer cost-effective, secure, and authorized connectivity among smart factories, workers, smart healthcare, transportation, and logistics. In addition, IIoT-based networks using wireless technologies require real-time monitoring, CPS, and smartphone-based IIoT applications. Moreover, smart IoT sensors monitor temperature, airflow, and humidity, keep safe historical records, and enable smoke and heat alarms. Similarly, smart industry servers, IIoT-based servers, and gateways play a crucial role in securing smart industry data and offer on-demand IIoT assistance to permissible subscribers. The top research trends in the IIoT domain consist of IIoT applications, network topologies, network architecture, communication protocols, and security challenges [7,8]. Soori et al. [9] present a review of the impact of IoT in the smart industry. Various applications of the IoT in smart factory environments are covered, like asset tracking, quality control, monitoring, energy optimization, etc. In addition, current challenges are identified to outline future directions. Although a significant amount of work has been conducted in the IIoT field, a comprehensive survey is required to identify the recent research trends in the smart industry.

### 1.1. Survey Contributions and Comparison with Related Work

In existing research, many surveys focus on the dependencies of IIoT components, security challenges, solutions, and characteristics. For example, the work presented by [10,11] focuses only on the research landscape of security challenges of IIoT but misses major attacks and their related countermeasures, while the current survey presents a comprehensive overview of attacks and countermeasures. On the other hand, [12] presents blockchain solutions for IIoT and attack taxonomies but does not provide a clear explanation for real-world mapping incidents. Comparatively, this study presents an extensive survey of recent research efforts and also provides real-world examples.

Similarly, in [13,14], a software-based and fog-based IIoT architecture is presented only. In contrast, this work not only describes IIoT network architecture but also presents comprehensive and updated literature on the layered structure of IIoT architecture. In a similar fashion, [15] proposed a fog cloud architecture between IIoT devices to briefly control the network traffic. However, the proposed architecture does not fulfill the security requirements; the current study presents an updated view of security-related needs and solutions for different IIoT sectors, security attacks, and threats.

The work illustrated in [16,17] explains the IIoT framework taxonomies to help researchers discover the security, network, and technology gaps but misses the security threats and their existing solutions. The current study briefly discusses attack taxonomies and their solutions. IIoT security research can significantly impact the industrial security process. Strong bonding between security and safety in IIoT is identified by [18]. Similarly, Ref. [19] propose a security solution and identify the security challenges in IIoT but lack taxonomy attacks and their solutions.

In comparison to the above-cited works, the current survey presents a comprehensive and extensive survey of IIoT attacks, weaknesses, and vulnerabilities, as well as measurements to overcome the identified security threats and challenges. In [20] the research gap of the manufacturing system between different layers of industry 4.0 is presented. Similarly, the survey [21] describes the scope of the study and challenges of intelligent factories extensively but does not explain the current modern applications of IIoT. In comparison to these, this survey presents an updated and comprehensive survey on IIoT applications, sensors, and smartphone applications. Table 1 provides the contributions of the current survey in comparison to existing works.

The contributions of this survey are not limited to the classification of privacy and security issues in IIoT; it also identifies the weaknesses, risks, problems, and challenges and provides future research directions to overcome these attacks. Many researchers have surveyed IIoT security challenges, attacks, and related countermeasures; however, the current work is more comprehensive than these studies. We present a state-of-the-art network infrastructure covering network topologies, network platforms, and network architecture based on big data and cloud computing.

### 1.2. Organization of Survey

Section 2 presents key components of the IoT-based smart industry together with the related technologies. In Section 3, the state-of-the-art network infrastructure is introduced, which includes a structural layer of IIoT architecture, IIoT network topologies, IIoT network platforms, and protocols. In Section 4, various IIoT application domains, smartphone applications, and sensor applications are presented. Section 5 presents IIoT-based security attacks and their countermeasures. In Section 6, research directions and future implementation of IIoT are discussed. Finally, in Section 7, open research challenges faced by technologists while implementing the IoT in the smart industry are discussed.

## 2. Major Components Related to IoT-Based Smart Industry

**Figure 2:**
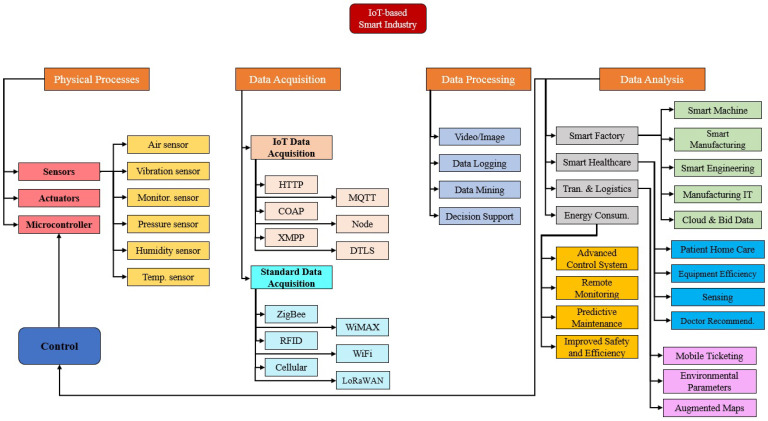
Key components of IoT smart industry.

The IoT industry comprises four elements, including data acquisition, physical structure, data analytics, and data processing, as illustrated in Figure 2. The most crucial aspect of the smart industry to avoid critical situations is the physical structure that controls all sensors, actuators, and devices. A sensor is responsible for various tasks, such as temperature monitoring, humidity monitoring, vibration sensing, current monitoring, pressure detection, etc. IoT gadgets, on the other hand, conduct various control functions, such as device identification, node discovery, and naming services. Any sensor or device controlled by a microcontroller can perform all these tasks. Each control activity can be remotely performed by any computer or remote device linked to the internet.

Data acquisition is the process of monitoring and analyzing various sensors, collected data, and hardware and is categorized into two sub-components: standard data acquisition and IoT data acquisition. The IoT data acquisition has six protocols: (i) node, (ii) message queuing telemetry transport (MQTT), (iii) datagram transport layer security (DTLS), (iv) constrained application protocol (CoAP), (v) extensible messaging and presence protocol (XMPP), and (vi) hypertext transfer protocol (HTTP). However, more protocols can be added or removed depending on the conditions and requirements of the designed system. The most commonly used protocols for standard data acquisition are ZigBee, Lora WAN, WiFI, mobile cellular networks, radio frequency identification (RFID), and WiMAX. Data processing includes several components, such as video or image processing, data mining, decision support systems, and data loading. Therefore, any feature can be implemented according to the system requirements and executed in parallel to offer additional services.

Data analytics aims to reduce costs by identifying more efficient methods of storing large amounts of data. Data analytics involves four sub-applications: smart factories, transportation and logistics, smart healthcare, and energy consumption in IIoT. Each device in a smart factory is connected to the internet and linked to actuators and sensors. The IIoT allows manufacturing devices to exchange data between service providers and users in smart factories [22]. Similarly, improved patient care, a faster and more accurate diagnosis, and more personalized treatment are also possible with the utilization of IIoT in healthcare [23]. Smart transportation-based IIoT helps to improve multiple devices and sensors, such as vehicle control systems, car navigation systems, traffic signal management systems, and speed monitoring systems [24]. In addition, the IIoT can reduce energy consumption, increase sustainable energy usage, and reduce the environmental impact of energy use [25].

## 3. IIoT Network Infrastructure

The IIoT network infrastructure is the backbone of the IoT for industries, and helps to connect many sensory, physical, and network devices to improve product quality and the manufacturing process, thereby playing an important part in the growth of IIoT. The infrastructure in IoT-enabled industrial network architecture comprises the industrial network platform, the network topology, and protocols.

### 3.1. Layered Structure of IoT-Enabled Industrial Network Architecture

**Figure 3:**
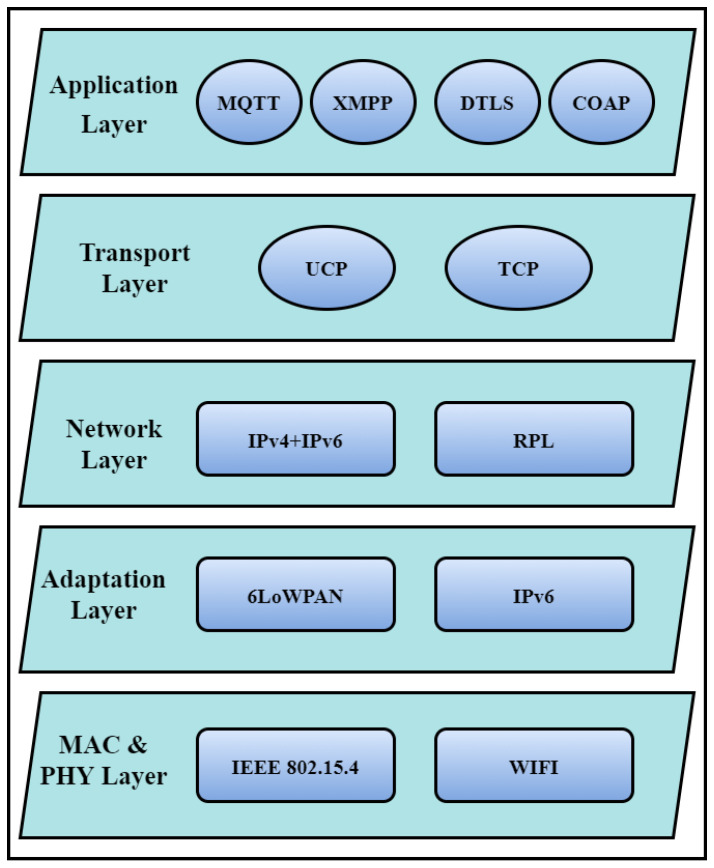
The 6LoWPAN layer structure.

**Figure 4:**
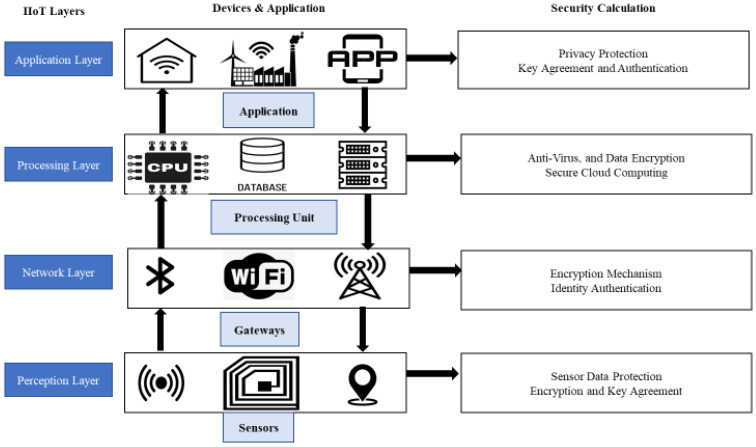
IIoT four-layer architecture.

The most important aspect of IIoT is the IIoT network, which connects devices, actuators, sensors, processors, cyber-physical systems, and production flow to make smart decisions [26]. The three-layer architecture, including the network layer, the perception layer, and the application or support layer, was initially presented by the researchers [27,28]. However, the three-layered architecture is weak from a security perspective and unable to fulfill the IoT network requirements [29]. For example, [28,29] points out several attacks on IIoT and various other security weaknesses. For that reason, the three-layer architecture has been shifted into a four-layer infrastructure (the perception layer, the network layer, the application layer, and the processing layer) to enhance the security of IoT networks [30]. After a comprehensive study of these four layers, two more solutions are IPv6 and 6LoWPAN, as shown in Figure 3. The support layer is the last stage of abstraction in the network architecture, which makes it more secure and robust. At this stage, communication protocols are used to keep track of several smart industry characteristics, like energy usage, cost reduction, and productivity enhancement. The network architecture includes sensors/devices at the first layer, communication devices at the second layer, data processing and storage at the processing layer, and smart applications at the fourth layer, as shown in Figure 4.

#### 3.1.1. Perception Layer

The perception layer is also defined as the sensor layer. It is a mixture of sensor and physical devices, global positioning system (GPS) modules, RFID, 2-D barcode, and closed-circuit television (CCTV) cameras [27]. It gathers data from all connected devices and sends them to the servers. In the industrial environment, devices are responsible for transporting raw items, monitoring production areas, and catching sensory data, industrial robots, automated guided vehicles (AGVs), and transporter systems. It is a sensitive layer and can be attacked easily. The security threats for the perception layer include node injection, tampering, eavesdropping, reply attacks, radio frequency (RF) interference, timing attacks, and node capturing [31].

#### 3.1.2. Network Layer

The network layer, also called data transmission, is responsible for receiving and transferring industrial information between physical objects, smart things, devices, sensors, networks, and servers using a wired or wireless medium [28]. It consists of protocols like IPv4, IPv6, WiFI, ZigBee, etc., and helps the connection between the perception (or sensor) layer. As a result, the network layer is susceptible to various attacks. Man-in-the-middle attacks (MITM), Sybil attacks, spoofing, denial of service (DoS), and sinkhole attacks are the riskiest and most well-known attacks on the network layer [31].

#### 3.1.3. Application Layer

The application layer is responsible for transferring IIoT applications from a connected device to the user. It works as a bridge between the end nodes and the network of IIoT, allowing them to communicate with approved software components [29]. The smart home, smart factory, and smart robotics are famous IIoT applications [32]. Securing the application layer is extremely challenging as security is a critical issue. Smart home applications are fragile to security issues because they are insecure from the inside and outside, which can introduce vulnerabilities. The security attacks on the application layer can be Trojan horses, malicious code attacks, cross-site scripting, and side-channel attacks [33].

#### 3.1.4. Processing Layer

The main reason for creating the fourth processing (or support layer) layer is various security issues in different layers of IIoT. The three-level architecture is not secure enough to pass data directly to the network layers; this layer overcomes multiple threats. The fourth-level architecture was proposed to overcome security issues in IIoT. Authentication is prioritized using passwords, pre-shared secrets, and keys and then sending collected data to the network layers. It contains databases and servers that can run various tasks like decision-making, storing vast amounts of data, and computer algorithms [13].

### 3.2. IoT-Enabled Industrial Network Platform

Big data analytics and cloud models have been included in the IIoT network platform.

#### 3.2.1. IIoT Network Platform Based on Big Data Analytics

**Figure 5:**
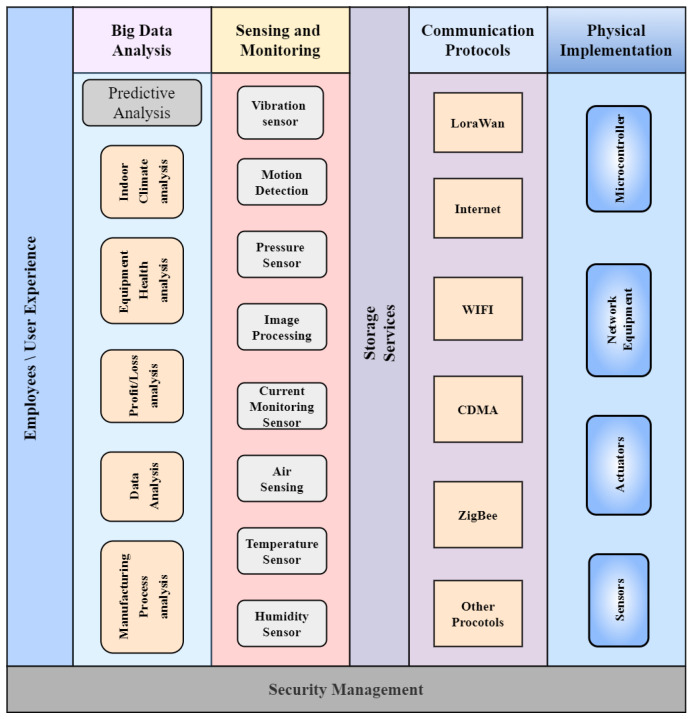
IIoT network-based platform on big data analytics.

Big data analysis collects essential and useful information from massive data and various types of data. The increasing deployment of sensors and IoT devices has resulted in a big data source in the IIoT. In IIoT systems, big data analytics is utilized for both functional and customer data. The big data-based IIoT network platform is shown in Figure 5. It is divided into six parts: big data analysis, employee experience, storage devices, monitoring and sensing, communication protocols, and physical implementation. This platform allows users to connect to the IoT backbone and collect data on equipment’s health monitoring, indoor climate, miniaturization, manufacturing process automation, etc. [34].

Employee’s Experience: The employee experience layer is created to benefit employees by monitoring equipment health and identifying temperature, humidity, air, pressure, and moisture-based indoor climate change. This identification helps industries to resolve production risks and increase income.
Predictive Analysis: Predictive analysis uses smart IIoT technology and market intelligence to make a smart environment. The key role of predictive analysis is to monitor, examine, and progress smart industrial technology for digital wakefulness. In addition, predictive analysis is used to check if the manufacturing process is working in the right direction without technical faults and risks. Based on manufacturing process management, different detection devices are used to identify indoor climate changes, equipment health, profit/loss estimation, and data analysis.
Sensing and Monitoring Analysis: The sensing and monitoring process is performed using various sensing and detecting equipment to store information about the manufacturing process. The sensing layer automatically analyzes the data collected from different resources. In addition, statistical analysis is performed on data received from sensors to actuate the production risks. Sensors such as vibration sensors, air sensors, temperature sensors, current monitoring, and humidity sensors provide crucial data regarding production units and help the smart industry run smoothly.
Storage Service: The data related to the smart industry are saved to perform future analyses to enhance manufacturing productivity appropriately.
Communication Protocols: Smart industrial data are collected and summarized in communication protocols. Therefore, the central pillar of IIoT analyzes and transmits data using different protocols. Third-party service providers like code division multiple access (CDMA), long-term evolution (LTE), or the global system for mobile communications (GSM) are no longer available. Researchers across the globe recommend ZigBee as the leading protocol for communication over long distances.
Physical Implementations: Several sensors, actuators, and microcontrollers monitor various IIoT applications. In addition, additional devices in the network such as routers, switches, and gateways are major components of the physical layer. This layer detects the whole environment and activates according to specified commands. The microcontroller works as a controller and performs network-related tasks and other functions handled by sensors and actuators.

#### 3.2.2. IIoT Network Platform Based on Cloud Computing

**Figure 6:**
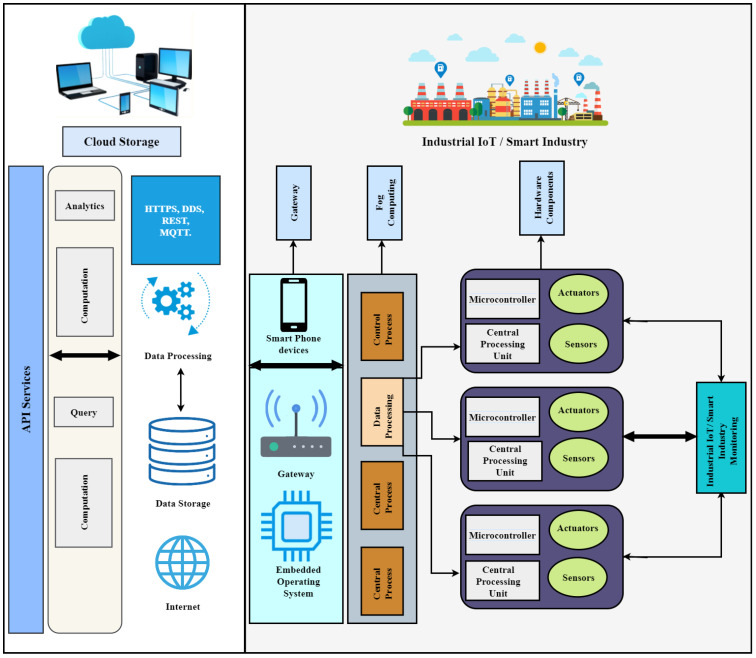
The cloud computing-based industrial IoT network platform.

Cloud computing delivers a huge amount of storage via large virtualized computers linked together. In addition, cloud computing and big data consist of the latest high-performance computing, IIoT technologies, service-oriented technologies, and cloud services [35]. Figure 6 shows the cloud computing-based IIoT network platform with four layers: cloud storage, gateway, fog computing, and hardware modules. The IIoT-related data, including manufacturing processes, indoor climate change, moisturizing, and smart industry marketing, are stored in the cloud. The networked infrastructure provides on-demand resources. In addition, online services and analytical resources are also stored and accessed via cloud computing [36].

Connecting a large number of devices to the internet for data sharing is not appropriate or safe. Instead, local gateways are designed to solve data-sharing problems by connecting all hardware devices and sensors for security, connectivity, and controllability. For example, a gateway in a production process or productivity unit controls a real-time manufacturing monitoring system and enhances automation. Similarly, fog computing allows the distribution of hardware components, cloud services, and the combination of available resources [37]. In addition, fog computing ensures real-time processing and reduces cloud computational load. The main objective of fog computing is to take advantage of both edge and cloud computing to maximize cloud computing resources and on-demand scalability.

Multiple sensors, actuators, microcontrollers, and a central processing unit are applied to components to sense and monitor different IIoT variables. Hardware components are delivered worldwide or locally and utilized to provide services or processes. A fast response time and the ability to exchange information are required for smart manufacturing deployment. MQTT and representational state transfer (REST) are two protocols that meet both the requirements of a quick response time and the ability to communicate information. Using a big data center, a distributed system is made more efficient for smart manufacturing [38]. It also divides large computations into simple and smaller jobs, such as controlling temperature, floor moisture, indoor climate, and production units.

### 3.3. IoT-Enabled Industrial Network Topology and Protocols

An IIoT network’s topology describes how different network elements of the IIoT are connected and provides an ideal smart industry scenario. Several IoT connection protocols are commonly used in the industrial field for the smart industry [39]. Using these protocols, employees/workers can communicate more easily and make effective decision-making for the smart industry to improve and monitor the manufacturing productivity of the unit.

#### 3.3.1. IoT-Enabled Industrial Network Platform

**Figure 7:**
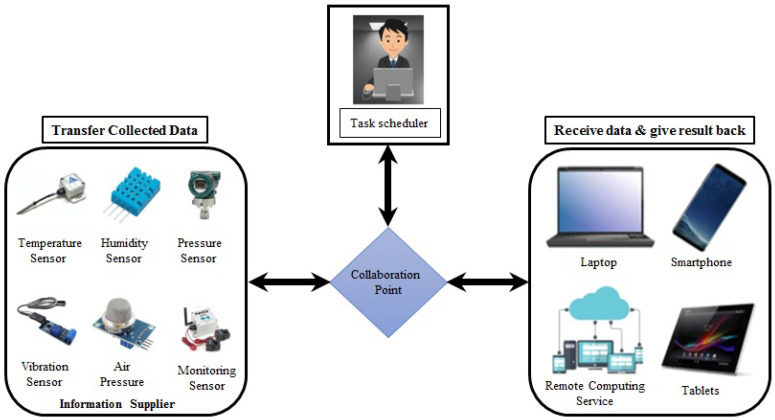
Conceptual designfor smart industry.

IIoT network topology provides a state-of-the-art network topology for the smart industry. IIoT network topology combines different sensors, actuators, and physical devices like pressure, temperature, humidity current monitoring, vibration, and water detecting sensors, as depicted in Figure 7. Moreover, the ideal scenario (conceptual design) for future smart industry solutions includes the help of storage devices such as laptops, tablets, smartphones, grid computing, and actuators [40].

**Figure 8:**
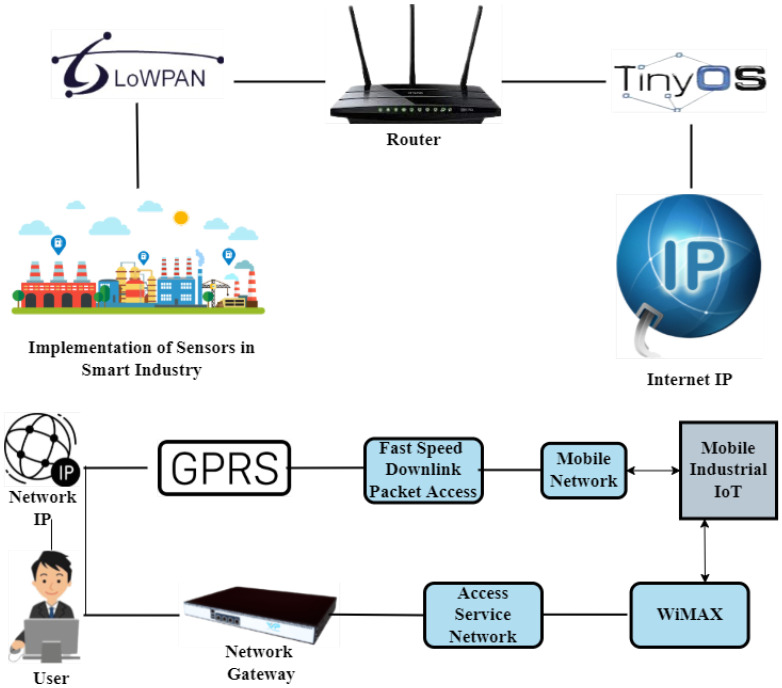
Remote monitoring topology in a manufacturing unit of smart industry.

A structural framework of IIoT network topology is given in Figure 8. The production unit and manufacturing sector are monitored with the help of different protocols and devices. Data from various sensors and devices are useful for aggregated data. Data are then processed and stored. Industrialists/employees can remotely monitor different manufacturing unit aggregations and analyses. Furthermore, topology comprises an appropriate network configuration for industrial video streaming [41].

**Figure 9:**
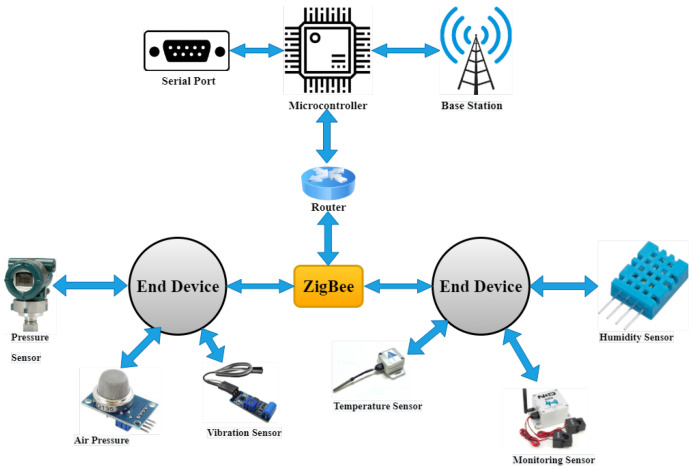
Low-power wireless sensor network topology.

Similarly, Figure 9 shows an interconnected network comprising internet protocol (IP), GSM, WiMAX, access service network gateway, and manufacturing units. A wireless sensor network is used to monitor (the manufacturing process) and control (power consumption) in many fields of the smart industry. ZigBee is used in network topology; the function of ZigBee is to transfer data or sensitive information through routers, sensors, base stations, and end devices. In addition, sensors like air sensors, temperature sensors, vibration sensors, humidity sensors, current monitoring sensors, and microcontrollers are used [42]. The router and microcontroller are connected directly to the end devices, and the microcontroller communicates with the base station through the serial port to examine the collected data. According to software monitoring, each end device is configured properly, and attached sensors are enabled. When the sensors are turned on, each device follows the router to connect in the designed way. End devices can connect to the WSN using the same key after validation. Sensor data are sent to the base station, which analyzes the information. Data are transferred through ZigBee to the controller or router when end-device sensors are read. The bidirectional communication via ZigBee is the main strength of this network topology.

#### 3.3.2. IIoT Communication Protocols

Various IoT communication protocols have been widely used in the smart manufacturing industry. Therefore, it is very useful to use smart manufacturing and monitor the rise in industry productivity by employing these protocols [39]. The most popular wireless protocols are ZigBee, Bluetooth, WiFI, MQTT, Lora WAN, mobile cellular networks, RFID, WiMAX, and LR-WPAN. Table 2 provides a summary of wireless protocols utilized in IIoT communication. These protocols are discussed from several aspects. For example, protocols are described with respect to operating frequency, IEEE standard, and transmission range. In addition, data rate, cost, and energy usage are also given. LoraWAN and WiMAX are better choices for long-range communication, but LoraWAN consumes less energy compared to WiMAX.

ZigBee: ZigBee technology is a low-data-rate, low-power consumption, and low-cost wireless networking protocol developed by the ZigBee Alliance for automation and sensor networks. The ZigBee network can contain many nodes in an industrial environment and connect them into a single control network [43].
Bluetooth: Bluetooth is a low-power, short-range personal area network (PAN). It was developed by Ericson but operated under the auspices of the Bluetooth special interest group (SIG), which created the Bluetooth standards (IEEE 802.15.1). Moreover, to close the energy efficiency gap between Zigbee and Bluetooth for no-streaming sensor node-type applications, the low energy standard for IIoT-based Bluetooth has been modified [44].
WiFi: In the current era of modern advancements, the availability of WiFi has become a necessity. WiFi stands for wireless fidelity, and was introduced by the Institute of Electrical and Electronics Engineers (IEEE) and is a communication standard for wireless local area networks (WLANs). WiFi operates on physical and data link layers. Furthermore, these standards operate at different bandwidths, ranging from 5 GHz to 60 GHz. The communication and manufacturing processes are discussed in [45].
MQTT: MQTT is a remote connection between two messages queuing telemetry transport protocols in the IoT. It is a combination of low-power protocols with high bandwidth efficiency. In the smart manufacturing industry, MQTT is utilized for monitoring and development. The use of MQTT to track, monitor, and investigate the manufacturing process and improve efficiency has been presented as a low-cost, web-based IoT solution [46].
Lora Wan: Lora WAN is a long-distance communication protocol designed for IoT and mobile-to-mobile (M2M) applications that provide a cellular-style, low-data-rate communications network. The primary goal of the Lora WAN protocol is to ensure interoperability across several operators in the IIoT [47].
Mobile Cellular Networks: There are many generations of mobile communication standards, including 2G, 3G, 4G, and 5G. Each generation of mobile phones has its own challenges and capabilities. For example, smart manufacturing based on cyber-physical manufacturing systems helps IIoT in automation, real-time monitoring, and collaborative control. Although 3G and 4G cannot meet the CPMS standard requirements, 5G can support IIoT [48].
RFID: RFID records data by assigning a unique number to each object. RFID systems comprise readers, hosts, and tags that receive and broadcast radio waves, also known as the communicators. RFID tags can be active or passive, and they come in a range of sizes and designs. Passive tags are less expensive than active tags and are more profitable. Tags have unique ID numbers and IIoT environmental information, such as moisture level, temperature condition, humidity, etc. In the IIoT, RFID monitors the manufacturing process [49].
WiMax: The data transfer rate of WiMAX ranges from 1.5 Mb to 1 Gb per second. However, technical advancements have improved the data transfer rate in recent years. Furthermore, WiMAX offers multi-access connectivity, including wired and wireless connectivity for fixed, mobile, portable, and mobile communication, used in IIoT [50].
LR-WPAN: In recent years, advancements in high-level communication protocols such as ZigBee have developed low-rate wireless personal area network (LR-WPAN) standards. LR-WPAN offers data rates ranging from 40 to 250 Kb per second. This standard’s key feature is that it delivers low-speed and low-cost communication services. It has a frequency band that ranges from 868/915 MHz to 2.4 GHz. LR-WPAN has been used in IIoT control applications and manufacturing monitoring systems [51].

## 4. IIoT Applications

**Figure 10:**
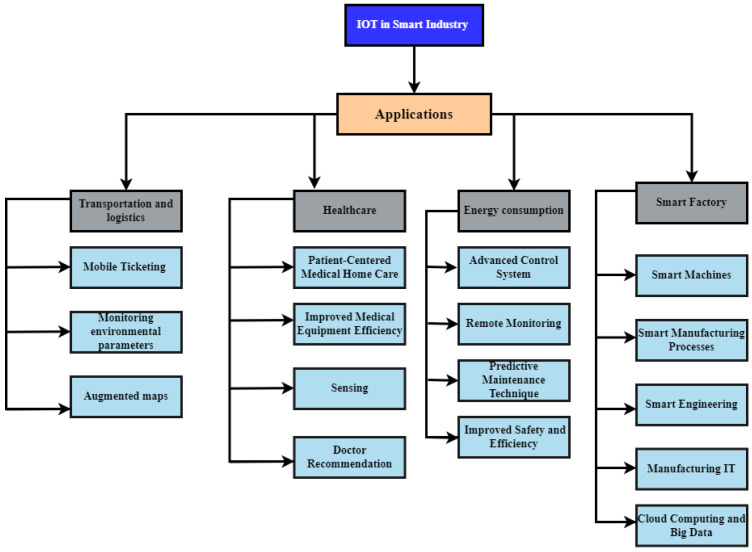
IoT applications in smart industry.

The IIoT system is used as an order in many fields, such as smart factories, healthcare, energy consumption, transportation, logistics, etc. There are three types of industrial applications: IIoT applications, sensor-based applications, and smartphone-based applications. The classification of IIoT-based applications is presented in Figure 10, which was created to examine the industry’s current IoT solutions.

### 4.1. IIoT Sub-Applications

Many IIoT applications have been used to create more effective resources for the fast growth of industry productivity. However, depending on the proposed industrial application, designers may trade off these goals to balance costs and benefits. Different types of industrial applications are discussed in the subsections that follow.

#### 4.1.1. Transportation and Logistics

The IoT is essential to the rapid growth of transportation, logistics, and industrial manufacturing processes [52]. Transportation and logistics companies can manage the real-time monitoring of the movement of physical objects from one place to another over the complete supply chain, including distribution, manufacturing, and shipping [53].

IIoT offers advanced technologies and solutions for the automobile and transportation industry [54]. IoT technologies have improved networking, communication, sensing, and data-processing capabilities for underused vehicles in parking spaces or on the road. IoT technologies make it possible to track vehicles, monitor their movement, and predict current and future locations. For example, a very effective and intelligent technology (iDrive system) made by BMW (Rolls Royce Phantom) uses different sensors and tags to monitor the environment, such as road conditions, to provide driving directions and trace the vehicle location [55]. Zhang et al. [56] built an intelligent monitoring system that uses RFID, sensors, tags, and wireless communication technologies to monitor humidity and temperature within refrigerator trucks. Tesla makes autopilot (advanced driving assistance system) vehicles that can monitor vehicles’ movement and be controlled remotely at any place [57]. IIoT technology such as RFID, autopilot systems, computer vision, and robotics have helped the transportation and logistics industry increase productivity and automate processes [58].

Mobile Ticketing: Smart transportation uses near-field communication (NFC) tags, a numeric identifier, and a visual marker [59]. Using IIoT technologies, consumers obtain information about various possibilities from the web services by passing their mobile phone over the NFC tag or directing their mobile phone toward the visual markers. The mobile phone obtains data from connected web services (stations, passengers, pricing, available seats, and type of services) and allows users to purchase equivalent tickets [60].
Monitoring Environmental Parameters: IIoT technology can help monitor our daily environment, like the temperature and humidity [61]. For example, food manufactured in a factory and traveling thousands of kilometers to reach customers must be monitored to reduce the risk of food spoilage. IoT-based advanced technologies, sensor technologies, and pervasive computing improve the productivity of the food supply chain [62].
Augmented Maps: IIoT-based applications make tourist maps, tags, and NFC-enabled smartphones available for browsing [63]. In addition, IIoT technologies help provide information on restaurants, monuments, hotels, and other locations relevant to users’ interests [64].

#### 4.1.2. Healthcare

The healthcare industry is benefiting greatly from IIoT applications [65]. They reduce cost and provide remote control of medical equipment, home-bound patient care, modeling, and monitoring [66]. As a result, hospitals benefit from smart equipment that decreases a patient’s waiting time and improves equipment performance. The popularity of mobile internet services has promoted the faster growth of IIoT-powered in-home healthcare (IHH) services [67]. Different health application domains can be helped, as mentioned below.

Patient-Centered Medical Home Care: Patient-centered medical home (PCMH) care is a simple solution to many problems faced by the healthcare industry, such as chronic disease management, overuse of emergency rooms, patient satisfaction, high medical costs, and accessibility [68,69]. The IIoT has completely changed the healthcare industry. The use of modern technology saves time and allows nursing staff to perform more work in less time, such as taking blood pressure without wasting time. IIoT devices can be utilized to collect patient data, upload it to the cloud, and have a doctor make a fast diagnosis and suggest appropriate therapy. Moreover, a doctor can make a timely decision for appropriate treatment. For example, Cambridge consultants’ flow health hub (FHH) IIoT home diagnostics can gather samples and promptly deliver blood pressure, cholesterol, and diabetes medication [66]. In addition, this method automatically alerts doctors that their patients need or want assistance.
Improved Medical Equipment Efficiency: The fast growth of IIoT technology gives doctors more useful information. With a concept known as medical device plug-and-play (MD PnP), IIoT allows modern medical equipment to be connected instantly. MD PnP is a cyber-physical system for medical devices [70]. The healthcare industry is affected by two sides of CPSs. The first involves discrete computer logic of various secured medical equipment in the cyber-world. The second is that it offers a complicated biochemical system that includes a patient-in-the-loop mechanism [71]. As a result, CPSs offer valuable data and reduce patients’ waiting times. Thus, CPS sensors provide real-time data to guide doctors in making the best decisions for their patients [72].
Sensing: Sensor devices provide valuable information on patient health and diagnosing patient disease [73]. In addition, the IIoT application domain offers telemedicine solutions such as informing patient welfare and monitoring patient health with advanced medical equipment [74]. Sensors are useful for both in-patient and out-patient treatment. In addition, wireless-based remote monitoring systems are generally employed to outreach to patients anywhere in the world through the employment of multiple wireless technologies paired with real-time bio-signal monitoring systems to capture the patient’s movements dynamically [75].
Doctor Recommendation: Today, choosing the right doctor online and getting an appointment is a tough job for patients. Patients have a big problem without real-time data and valuable information about professional doctors [76]. In this capacity, IIoT-based applications have developed a doctor recommendation system to get an online appointment with a doctor [77]. In addition, recommendation systems are still a hot topic in machine learning, image processing, and data mining [78]. The sensor data received from patients, feedback for qualifying doctor suggestions, and doctor appointment policies have been updated in the doctor recommendation system [38].

#### 4.1.3. Smart Factory

Smart factories utilize IIOT technologies to connect machines to humans (M2P) by using controlling devices like operation devices, field devices, mobile devices, and so on [39]. The purpose of smart factories is to provide smart products, services, and feedback to the client. Furthermore, cloud computing and big data are used to build smart factories’ manufacturing processes, hardware, and software [79]. Wang et al. [80] present a smart factory design that describes how to link cloud computing, an industrial wireless network, and workstations with smart shop-floor devices. Smart machinery, smart manufacturing, smart engineering, manufacturing information technology (IT), cloud computing, and big data are the essential components of a smart factory [40].

Smart Machine: A smart machine combines an autonomous, networked system, sensors, processing capabilities, and communication devices in IIoT [81]. Smart machines have also been linked to other field devices and humans and can work remotely. In addition, smart machines use IIoT to perform self-operability, self-maintenance, and self-awareness [16].
Smart Manufacturing: The IIoT directly impacts the manufacturing industry by merging cyber-physical production systems and the IoT, resulting in smart manufacturing, which connects the practical and physical worlds [41]. The smart manufacturing process is automated, efficient, and effective, and its real-time performance is one of its key characteristics [82]. Smart manufacturing processes require industries to dynamically fulfill customer requests based on the interconnectivity provided by the IIoT to manage personalization [44]. Furthermore, customer feedback plays a vital role in manufacturing [83]. As a result, both the cyber-physical production system and the IIoT concepts are integrated into the smart manufacturing concept [84]. IIoT consists of smart sensors that can send information about machines, fleets, and components and monitor the production system [85].
Smart Engineering: Smart engineering in smart factories creates product engineering, product design, and product development [86]. Big data analytics are generally employed to attain continuous feedback, providing a more effective engineering process in IIoT, dispensing efficient optimization, and improving productivity.
Manufacturing IT: Manufacturing IT refers to smart factories’ information technology infrastructure [79]. Manufacturing IT involves the production system’s algorithms, software, and hardware infrastructure, such as sensors and actuators that offer smart monitoring and control of physical devices. In addition, IIoT enables production management systems to integrate many technologies and maintain all data generated during manufacturing.
Cloud Computing and Big Data: The latest high-performance computing, IIoT technologies, service-oriented technologies, and cloud services are part of cloud computing and big data [35]. In addition, cloud computing and big data built a business model for the manufacturing industry, creating smart factory networks that support productive collaboration and helping it adjust product innovation with business policy [36]. Cloud computing fulfills customers’ requests for services, including product design, management, manufacturing, and testing. Moreover, trends in smart manufacturing, innovation, and future methodologies focus on the cloud, the CPS, and the IoT [37]. For example, the design of smart manufacturing has been reviewed by Saldivar et al. [87]. In addition, Rugman et al. [88] explain the benefits for the manufacturing industry and highlight the latest technologies, such as big data analytics, autonomous robots, cyber security, system integration, cloud computing, augmented reality, simulation, and additive manufacturing.

#### 4.1.4. Energy Consumption

IIoT technologies have modified the energy sector, and efficient sensor monitoring systems have decreased factory energy usage [89]. Therefore, the industrial energy system is an important component of the IIoT. In addition, IIoT technologies have increased the performance of new energy systems. Furthermore, a new energy system increases environmental security [90]. The study [91] provides an energy-efficient design for energy-constrained mobile devices. The authors consider multiple-antenna access points for radio frequency energy harvesting using non-orthogonal multiple access (NOMA). For obtaining a better system performance, the communication protocol comprises four phases. Results indicate a 3 to 30% improved performance of successful computation probability. When compared to traditional energy systems in IIoT, the new energy system has the following characteristics:

Advance Control System: The management and control of old energy systems require many workers, whereas the new IIoT energy system requires less labor [92]. Furthermore, the effective application of new technologies in connectivity and interoperability improves system operability. The latest communication and information technologies have a tremendous change in the IIoT energy system, such as big data analytics, software-defined machines, and smart sensing [13]. These new technologies have continually been improving the system’s operational performances.
Remote Monitoring: Old energy systems needed a large amount of labor to run them. In contrast, the new energy production systems use remote monitoring systems to build a safe environment in IIoT. The IIoT system utilizes communication and sensor technologies to operate the production system remotely. Remote monitoring technologies can help the energy industry enhance its production performance while also reducing the risk for workers [93].
Predictive Maintenance Technique: Energy production systems in the IIoT hold data analytics and big data to generate predictive analytics information to help prevent unplanned downtime and major losses and minimize the risk of a complete shutdown [94]. However, the energy production industry faces a big problem in maintaining good conditions of the equipment.
Improved Safety and Efficiency: Different security policies exist for IIoT risk management and system control security principles [95]. In addition, the IIoT energy system can detect faults and energy consumption of multiple components through continuous monitoring and real-time data processing. As a result, the system can reduce serious and dangerous incidents and unnecessary losses and increase overall energy efficiency [42].

### 4.2. Smartphone Applications Solutions for IIoT

**Figure 11:**
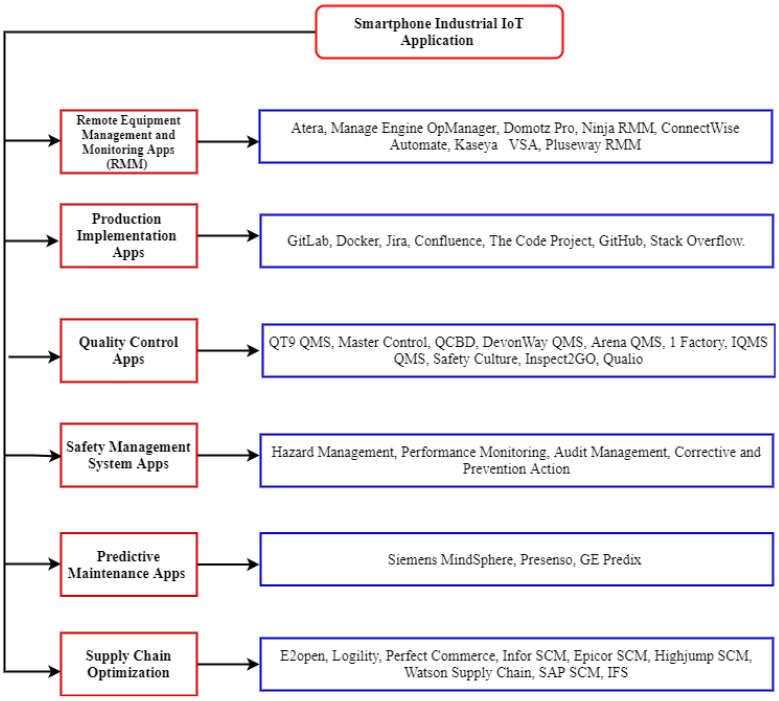
Smartphone applications for Industrial IoT.

Smartphone applications (apps), an innovative technology that combines electronic devices, are used to drive IoT. Smartphone applications have been created for the industrial sector. In [96], the authors present smartphone apps that provide industry solutions. The categorization design of smartphone apps for the smart industry is shown in Figure 11. All of the smartphone apps are presented in Figure 11, with a brief description of each app. Developers from all over the world have built many e-industry apps; this survey highlights a few selected apps based on their popularity. Apps are divided into different categories:

- Remote Equipment Management and Monitoring Apps: Apps used to manage and monitor the equipment remotely, like Atera, Domotz Pro, etc.
- Production Implementation Apps: Apps providing platforms for administrators for production control.
- Quality Control Apps: Apps aiming to provide quality control for single and multiple software, while others provide long-term tracking solutions.
- Safety Management Apps: Apps that focus on providing different kinds of security controls like hazard management, audit management, and corrective and preventive action.
- Predictive Maintenance Apps: Apps that provide predictive tools for predicting asset maintenance.
- Supply Chain Optimization Apps: These apps offer platforms to optimize supply chain operations.

### 4.3. Sensors and Devices in Industrial IoT

Everything needs to be automated with fewer human resources by using less time in today’s world. The sensor is one such device that can fulfill a specific need by detecting and responding to the same input from the current physical environment [96]. Users configure some settings on sensing equipment to execute tasks without using human resources. Users set some settings over sensing devices to complete their jobs without the involvement of human resources in some major IoT sensors. Temperature, humidity, pressure, current-monitoring, vibration, and water-detection sensors are important IoT sensors. Sensor-based industrial applications are shown in Table 3. The table describes various industrial sensors used in IIoT, including humidity, pressure, temperature sensors, etc. These sensors are discussed with their attributes and shortcomings.

## 5. IIoT Security Threats

**Figure 12:**
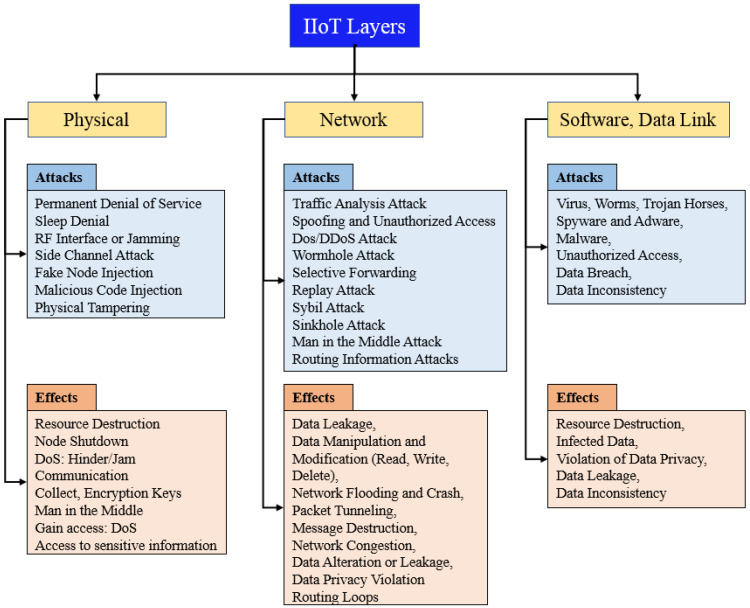
Industrial IoT attacks.

Many researchers have briefly discussed IIoT security and privacy threats; this work highlights the attacks on each layer of the four-level architecture of IIoT and provides its countermeasures equally [105,106,107]. For example, [108] analyzed the security technology trends for smart factories regarding IIoT. Security protocols are discussed from an automation and manufacturing industry point of view. In addition, recent IIoT-related security solutions and technological advancements are discussed in particular. This research reviews the literature on IIoT security attacks and presents countermeasures in the following sections. The IIoT security attacks are summarized into three parts: physical attacks, network attacks, and software and data link attacks. The effects of these attacks on the four-level IIoT architecture are shown in Figure 12 [109]. A thorough analysis of IIoT and Ir 4.0 security protocols is presented in [110]. Recent research developments are analyzed, particularly those involving blockchain. In addition, the challenges of implementing cryptocurrency are also explored.

### 5.1. Physical Attacks

These attacks include IoT hardware and physical devices, and attackers remain close to the device or network of the system [109]. Physical attacks harm the user’s sensitive information like passwords. A description of the different types of physical attacks is provided here.

#### 5.1.1. Permanent Denial-of-Service

Permanent denial-of-service (PDoS) is a denial-of-service (DoS) attack that can harm the hardware of IoT devices [111]. Phishing is another term for this type of attack. PDoS disables a system’s functionality and firmware [112].

#### 5.1.2. Denial of Sleep

An attack prevents battery-powered sensor nodes from entering sleep mode, causing network performance issues [113]. In addition, it is also possible to target networking devices to prevent communication and block traffic resulting from denial of sleep attacks.

#### 5.1.3. RF Interface/Jamming

Through RF, the attacker can create and transfer noise signals to distract communication and DoS attacks in RFID nodes [114]. In general, the function of a radio frequency interface (RFI) attack is to divert the user to get themselves connected to a fake rogue base station (RBS) while abandoning the legitimate operator signal. These attacks are based on a suitable combination of targeted jamming signals.

#### 5.1.4. Side-Channel Attack

A cloud service provider secures IoT security, and industries are aware of that kind of attack. Encryption keys are the focus area of the attacker; these keys encrypt/decrypt users’ sensitive data [115]. The side-channel attacks (SCAs) are generally grounded on power consumption, electromagnetic, timing, and laser-based attacks. Contemporary IoT technologies include mechanisms to prevent these attacks through the use of cryptographic security.

#### 5.1.5. Fake Node Injection

Many nodes work together to create a fake report and inject it between the control data flow and the system’s network [116]. Later on, faulty information is provided by the applications that impact the effectiveness of the IoT platform.

#### 5.1.6. Malicious Code Injection

The malicious code injection (MCI) functions to force users to act unknowingly as their information is stolen, usually through cookies. Hackers can target users by sending malicious links via social media sites or email. After clicking the link, the user is redirected to an untrusted server, reflecting the attack on the user’s browser [117].

#### 5.1.7. Tampering

The attacker has physical access to modify the device interface like communication devices and RFID [118]. The function of tampering is to manipulate application information that is transferred between server and client. In this case, every bit of information is sent to the application through a POST request.

#### 5.1.8. Countermeasures for Physical Attacks

Many approaches have been published to overview the critical research work on countermeasures [119,120]. For example, Sicari et al. [121] proposed solutions against network smart object (NOS) middleware and REATO, which is attacked by DoS in the IoT environment. This technique involves sending HTTP connection requests to NOS and receiving authentic information in return. Hsiao et al. [122] developed a support vector machine (SVM) technique to organize a model that avoids security risks in IoT applications. The author of [123] predicts the results with the help of SVM to show how effective it is in the medical field. SVM highlights resource depletion as the leading cause of sleep denial attacks. The author of [124] proposed a model named smart-fusion2 SoC, which prevents attacks like jamming. The author also presents the architecture model of (Cute Mote) for better performance and energy production. A side-channel attack is dangerous for IoT devices; a technique known as physically unclonable function (PUF) that protects from side-channel attacks is shown in [125]. Saivarun et al. [126] provide a solution to monitor a smart industry and alert in case of any threats. For data analysis from the IoT sensors, cloud service is utilized. Porambage et al. [127] describe the PAuthKey protocol, which builds implicit certificates connecting the peer sensor node and end-users. This mechanism creates a security boundary with the help of sensor node protection from different attacks like fake node injection. Deepa et al. [128] highlight various attacks (tampering and malicious code injection) that access users’ sensitive information. PUF-based authentication prevents these attacks. A list and short description of countermeasures for physical attacks are shown in Table 4. Each physical attack is discussed concerning its possible impact on the network. In addition, probable countermeasures and solutions are also suggested for each attack.

### 5.2. Network Attacks

The network layer is a sensitive layer that can easily attack and damage network devices [129]. Information integrity and confidentiality are generally threatened by the general security issues of the network layer. The description of attacks is as follows:

#### 5.2.1. Traffic Analysis Attack

The attacker gains access to network-sensitive information without entering into the network [130]. In addition, various forms of malicious behavior can be launched by the attackers such as back attacks and hop-by-hop tracing to attain the precise position of the key nodes.

#### 5.2.2. Spoofing Unauthorized Access

The attacker acts on behalf of another person and gains access to sensitive data through RFID signals. In this type of attack, attackers quickly change the IP address packets and send malicious code [131].

#### 5.2.3. Distributed Denial of Service Attacks

The reverse of a DoS attack is a distributed DoS (DDoS) attack, which brings down a server or network system. DDoS can target a specific flooding message with a node attack [132].

#### 5.2.4. Wormhole Attack

Tunnel packets are moved from one place to another over a low-latency link created by an attacker [133]. The primary objective of a wormhole attack is to dislocate the flow of traffic and the network topology. This type of attack is executed by producing a tunnel between two attackers and transmitting all the traffic toward the targeted node.

#### 5.2.5. Selective Forwarding

The attacker can send malicious code messages and drop them into one network node, but data cannot reach its location [134]. In addition, the selective forwarding attacks are concealed by the normal packet losses, which complicate the attack detection. Hence, it is generally stimulating to identify selective forwarding attacks and enhance network efficiency.

#### 5.2.6. Replay Attack

This attack is the leading cause of the DoS attack. The attacker often sends signed packets with wrong values to the same destination [135]. The increased risk of replay attacks is because the attacker does not need high-level hacking skills to attain information by decrypting after capturing the information. In general, attackers are successful by resending all the information.

#### 5.2.7. Sybil Attack

The Sybil attack targets the compromisation of the privacy of users’ information. In this type of attack, the hacker can breach the IoT-distributed cloud storage nodes and become a participating member of the network. In addition, the compromised users let the hacker detain some amount of distributed storage [136]. An attacker creates a false identity and authenticated access to WiFi.

#### 5.2.8. Man-in-the-Middle Attack

This type of attack infects both the wireless and wired users present on the IoT network. A practical scenario of this attack can be related to a football case where the third player tried to intercept while the other two players tried to pass it. Hence, this type of attack threatens the overall network communication. An attacker drops eavesdropped messages between two communication IoT devices and accesses their sensitive information [137].

#### 5.2.9. Routing Information Attacks

These attacks are produced through modification in routing data. These attacks are highly damaging to the network as they input the wrong routing table entries into the routing table. Then, attackers send malicious messages and leak the network’s routing information [138].

#### 5.2.10. Countermeasures for Network Attacks

This section focuses on published works to overcome the attacks faced in the network layer. Liu et al. [139] provide a framework for privacy-preserving traffic obfuscation and defenses against traffic analysis attacks in various IoT applications. The results show that network utility cost and privacy protection are better than others. Farha et al. [140] describe a secure static random-access memory (SRAM)-PUF-based entity authentication technique for IoT device authentication. This technique uses challenge-response pairs (CRPs) to overcome the challenges and increases the response time of SRAM cell values. The value result shows that this scheme is better for resources constrained with a low memory size in IoT devices.

The study [141] proposed a secure routing protocol for low power (SRPL), which is resistant to skin-hole and routing attacks and prevents malicious code by using secure authentication hash values. Tao et al. [142] proposed the great-alternative-region (GAR)-based approaches that overcome the physical attacks problem. Intrusion detection systems (IDS) create attack detection techniques to prevent IoT devices from threats [143,144]. IDS protects from sinkhole and wormhole attacks. Djedjig et al. [145] introduced the measure-based RPL trustworthiness scheme (MRTS), which benefits energy usage, a trust-based routing metric, packet delivery ratio, consistency, and node rank changes. Haripriya et al. [146] proposed a secure MQTT system to avoid intrusion detection. It can protect IoT applications from DoS attacks. The experiment results show that the proposed scheme detects malicious nodes between IoT devices better than existing techniques. Thus, MQTT-based authorization shows better performance and privacy protection systems in IoT networks [147]. Karati et al. [148] proposed that encryption is perfect for data confidentiality and the authenticity of data transmission in IIoT systems. On the other hand, Yin [149] proposed two frameworks. Firstly, software-defined IoT (SD-IoT) uses IoT devices and gateways, and second, an algorithm is used to prevent and protect from DDoS attacks. The results of both models show that the algorithm presents better performance countermeasures, as shown in Table 5. It provides an overview of network attacks launched on the IIoT network. Various types of attacks are summarized along with their possible damage and probable countermeasures.

### 5.3. Software and Data Link Attacks

With the rapid development of IoT/IIoT, attacks on IoT devices, applications, software, and networks have also increased [7]. In the following, a description of attacks faced by the IoT world is provided.

#### 5.3.1. Trojan Horses, Virus, Adware, Worms, and Spyware

Contemporary IoT appliances include programmable embedded systems. Moreover, most IoT devices run complex software for general purposes. Therefore, such devices are always at a security risk. For instance, a computer can become infected through the internet by a virus or Trojan. These are malware software that gain access to a user’s system without permission and spy on sensitive information. Then, they perform a malicious task and are ready for further attacks [150].

#### 5.3.2. Malware

In general, this type of attack is called a cyber attack in which malicious software executes unauthorized actions on the targeted user node. The malware virus is launched in various forms and encapsulates various forms of attacks, for instance, ransomware, spyware, command, and control. Malware corrupts data centers or the cloud and destroys sensitive data stored in IoT devices. Security firewalls and anti-virus are two possible ways to prevent malware [151].

#### 5.3.3. Data Breach

A data breach is a general issue where the sensitive information is leaked. The protected information no longer remains trustworthy and is linked to an untrusted environment. In general, an information breach occurs as a result of a hacker attack or inside a corporation by an individual or a previously employed individual, leading to exposed data. The data breach is an attack to gain access to users’ sensitive information [152].

#### 5.3.4. Data Inconsistency

In general, issues related to data inconsistency or redundancy commonly occur in IoT devices. Data inconsistency leads to the complication of multiple tables within the same database creating database issues. The data become inconsistent and redundant, having various inputs for the same entries. In addition, data inconsistency is usually compounded by redundancy. For example, multiple tables have the same data but different inputs [153].

#### 5.3.5. Countermeasures for Software and Data Link Layer Attacks

Researchers highlight various solutions that help to prevent different attacks. For example, Batra et al. [154] proposed two solutions using secure security solutions like a lightweight IoT-based framework wireless network system (WNS). The proposed security solutions provide outstanding results. In addition, high-level synthesis (HLS) presents a secure high-level architecture protected from malicious activity [155]. Latif et al. [156] proposed a lightweight prediction model based on a random neural network (RaNN). The prediction accuracy of this model is better than other models. The accuracy of the proposed approach is better compared to IoT-based machine learning schemes. Zheng et al. [157] use an attribute bloom filter to cover all the characteristics in the access control system and present a privacy-preserving attribute-based online–offline encryption (ABE) for medical data exchange. As a result, only medical users encrypt the message to the server and decrypt the message using access control technology. Jiansheng et al. [158] propose two privacy-preserving technologies: attribute-based encryption (ABE) and blockchain-based access control data privacy schemes for IoT systems. The proposed scheme is more secure and efficient and solves authentication challenges.

A data breach is a harmful attack to gain access to users’ sensitive information. To overcome these threats, the author in [159] propose two-factor authentication dynamic privacy protection (DPP) and improved secure directed diffusion (ISDD). The authors utilize dynamic programming to obtain better results for privacy protection security in IoT devices. Furthermore, Gope et al. [160] proposed a two-factor authentication approach based on PUFs that were both privacy-preserving and lightweight. These authentication models show higher performance and security against attacks on IoT devices.

Song et al. [161] investigated IoT attacks and proposed a chaos-based privacy-preserving cryptographic system as well as a message authentication code (MAC) to protect data transmissions within a smart home. The suggested chaotic system generates symmetric keys using a logistic map to protect data transmissions and ensure integrity. Furthermore, in [162], the authors present secure blockchain-based framework solutions for the image encryption algorithm to prevent different attacks. A brief overview of the discussed countermeasures is provided in Table 6. This table presents trojan horses, malware, unauthorized access, data breach, and data inconsistency attacks, which are launched on the link layer. These attacks can cause resource destruction, data infection, privacy violation, data leakage, and data inconsistency. Possible solutions to avoid these attacks include high-level synthesis, neural network frameworks, privacy-preserving and blockchain-based solutions, and two-factor authentication.

## 6. Research Directions and Future Implementation

This survey presents an overview of the taxonomy of IIoT security attacks and their countermeasures and challenges. The IIoT sector is facing multiple challenges, including high traffic, regular dataset updates, data confidentiality, lack of IoT dataset availability, a complicated network topology, security, and privacy [150]. These challenges heavily influence the IIoT system and manufacturing processes. The IIoT sector faces various intrusion and application-specific flaws as well. These factors include a lack of maintenance in IIoT appliances, accidental vulnerability, and major financial, technical, and human loss [12]. This survey also presents challenges and solutions to ensure security regarding IIoT implementations for future applications.

### 6.1. Blockchain and 5G Technologies

Blockchain and 5G technologies are expected to play a major role in developing future IIoT. For example, Ling Liu et al. [163] investigated how 5G can work for efficient energy management in the IIoT. In [164], the author investigated how blockchain can be used for shared data storage in the IIoT. However, in this survey, a four-layer architecture and strategy are presented for combining IIoT with other technologies. This architecture considers different IIoT deployments, security requirements, compatibility, timeliness, scalability, and other related factors [165].

### 6.2. IIoT Integration with Security Systems

There is no integrated model for edge and data-level security in IIoT devices [166]. Furthermore, no model is a suitable fit for various automatic functions of IIoT. As a result, the compatibility and verifiability of the system integration should be further investigated.

### 6.3. IIoT High-Power Secured Communication Model

Data transmission through a public network causes vulnerability and increases security risks since IIoT devices are not secure. Data access problems can be reduced by introducing a high-power secured protocol [167].

### 6.4. Detective and Preventive Measures

A lack of preventative measures causes virus and injection attacks. The majority of attacks are detected through analysis rather than prevention. As a result, some effective measures for detecting assaults and preventing them from happening again are required. There is a major need to introduce sophisticated malware detection technologies to protect IIoT devices from attacks [168].

### 6.5. Advanced IIoT Support Architecture

Advanced IIoT architecture must be created for platforms with low feedback latency. Furthermore, few security systems in IIoT can support heterogeneous platforms, and backward compatibility has also been a research challenge [169].

### 6.6. IIoT Security Authorization Models

Authorization models with double-layer validation should be available in the future to improve IIoT security [170]. Furthermore, user modification should be provided in a way that is consistent with all application frameworks. Deep learning algorithms and a game-theoretic approach can build an application-based security measure for sensitive data. Table 7 shows some leading technologies from well-known companies along with their future trends and directions. These companies offer different solutions for IoT networks. IBM provides AI-supported visual inspection of components for quality control workers. Intel enables the deployment of smart factory solutions. Samsung provides an SDS platform to interconnect various IoT devices.

## 7. Industrial IoT Challenges

The key motive for manufacturers, healthcare providers, utility companies, industry automation, and agricultural producers to deploy IIoT is to boost production and efficiency. IIoT has various technical challenges, including efficiency, security, privacy, connectivity, interoperability, scalability, flexibility, and resource management. A few critical challenges that need to be resolved are discussed here.

### 7.1. Energy Consumption and Management Schemes

Industries are the greatest electricity users in a country, demanding energy-efficient power management methods. Some IIoT applications run on batteries for years, and this costly energy consumption needs low-power sensors and actuators that do not require batteries. Therefore, energy consumption affects network life, robotic devices, sensors, and actuators, so is an essential factor of IIoT. In addition, data packets continuously exchanging results is a leading cause of energy consumption. LPWAN technology enables low-power and low-cost operation in energy efficiency and consumption systems [178]. Although there are many technologies for energy consumption and efficiency, energy harvesting is a promising and emerging approach for IIoT [179]. Solar, radiofrequency, and thermal energy harvesting techniques provide low-power, availability, and low-cost benefits and should be enhanced further to increase efficiency [180].

### 7.2. Energy Optimization

Energy optimization is an area of increased research attention in IIoT. The lifetime of IIoT systems is affected by limited resources, so energy-optimized schemes are significantly important [179,181]. IIoT comprises various sensors and devices that require substantial amounts of energy [182]. It also leads to a higher carbon footprint. Energy-efficient communication is the need of the hour for IIoT systems [183]. Similarly, since IIoT devices also involve computation, energy-efficient computing is also needed [184].

### 7.3. Data Confidentiality

The IIoT collects increasing amounts of data; for example, cloud services [185] use processes and meta information for control and optimization. Customer information and company secrets are among the data that must be kept safe from unauthorized access. The main problem is maintaining confidentiality while allowing approved IIoT services to process and analyze the data.

### 7.4. High Connectivity in IIoT

The IIoT’s major advantages are strong connectivity between IT, operating technology, and the internet, which allows for more efficient and adaptable industrial production [186]. However, the separation and isolation of IIoT devices based on their functionality and preventing unauthorized access have become increasingly challenging. Nevertheless, the National Institute of Standards and Technology (NIST) [187] network segmentation is a reasonable option for securing industrial control systems (ICSs), controlling high connectivity, and requiring further investigation for IIoT applications.

### 7.5. Network Latency

Network latency challenges increase as the number of shared devices increase in IIoT [188]. Fog computing or edge computing is used to reduce latency in the network. Fog or edge computing applications require an end device or to be pushed towards the network edge to minimize response time and latency. The edge computing paradigm is built on the cloud computing paradigm by relocating services that are not fit for cloud execution to end devices.

The paradigm shift lowers overall network latency while improving the quality of service (QoS). Fog computing is ideal for IIoT systems that require low-latency and real-time performance [189]. Cloud offloading is another model in which computation-intensive tasks are uploaded to the cloud for quick and predictable execution [190]. Non-real-time apps are placed on the cloud, whereas latency-sensitive applications are executed in local networks using machine-to-machine communications [191].

### 7.6. Limitations of Sensors in Industries

IIoT uses many sensors to increase efficiency and improve product quality, and such sensors are temperature, ethnography, motion, sound, laser scanner, radar, color, light, and X-ray [192]. Moreover, recent advancements in microelectronics linked with improvements in solid-state sensors have drastically lowered the complexity of simple sensors and are less of an issue for the future. Instead, the challenge has been making them more selective in congested, noisy, and complicated conditions. Applying algorithms related to fuzzy logic guarantees to reduce such issues for future applications [193].

### 7.7. Co-Existence and Interoperability

Many subsystems and external systems would connect in the IIoT, resulting in interoperability problems [194]. For example, a smart industry is linked to an external smart grid, a production plant is linked to the WoT service, and the factory’s production system is linked to the same factory’s storage system. In addition, a variety of sensors and techniques would be used. As a result, integrating systems and sensors, as well as interoperability protocols, becomes more challenging. In the future, IIoT devices based on detection, identification, and reduction in external interference can achieve successful coexistence. Because many of the tasks (such as those in a production setting where actuators are required to initiate actions) are time-sensitive, the integration and interoperability must be perfect to offer excellent performance.

### 7.8. Scalability

The scalability of machines and factories becomes a basic problem in IIoT as the number of linked devices increases [195]. The scalability problem in the IIoT is caused by three factors:

i. Scalability of data. The increasing number of sensors in IIoT creates a considerable amount of sensing data continually. As a result, the process required for industrial control applications, such as motion-control applications, is typically very high.
ii. Furthermore, the high-frequency data scalable combination affects the system’s scalability. For example, control systems are usually controlled independently in traditional industrial approaches and do not scale. As a result, enabling heterogeneous devices and approaches to communicate becomes challenging.
iii. Collaboration. Scalable management becomes a challenge for heterogeneous devices. The horizontal and vertical integration of numerous industrial components and systems presents a non-trivial management and maintenance challenge to system administrators. As a result, to achieve scalability, current management technologies must be integrated into the system management process.

High-frequency data overcome the problem of data scalability by reducing bandwidth and improving system scalability. Furthermore, when multiple systems integrate and collaborate, combination scalability requires the lowering of the human effort in configuration. As a result, several communication protocols such as data distribution service (DDS), advanced message queueing protocol (AMQP), and MQTT have been proposed to overcome scalability in IIoT [196].

### 7.9. Fault Detection and Reconfiguration

The chances of failure rise as the IIoT system becomes more automated and more heterogeneous devices are used [197]. Some common examples include device failure, delayed communication, and connectivity issues. An efficient IIoT system must be robust, identify and endure common errors, and detect problems in real time. Advanced defect detection algorithms have been used at the hub, gateway, or middleware to coordinate various machines and devices. To detect problems, accuracy and timeliness are also essential. A single malfunctioning object can take control of a whole manufacturing or industrial process, resulting in financial, energy, and other resource losses. Without the need for human involvement, a faulty network of sensors or equipment should reconfigure itself. If a sensor stops working due to a fault, it can be put to sleep until it is replaced, and the sensor network configurations can be changed. In this manner, it ensures robustness while also saving energy.

### 7.10. Long-Lived Components

Consumer IoT devices have a far shorter lifespan than IIoT devices [198]. This increases the need to consider application and communication security during device creation and, more significantly, to update the software after devices are deployed. However, this problem is not limited to newly deployed devices; it directly impacts existing devices delivered with few or no security features and a complex upgrade method, despite being supposed to be used for decades. Furthermore, as the IIoT becomes more connected, the potential of security breaches rises, especially if formerly isolated older components become part of the network.

### 7.11. Security and Privacy Challenges

IIoT requires security assurance [199]. However, it becomes difficult to maintain the authenticity and data secrecy of the system when distributed sensor nodes, actuators, and machines are coupled in a production system. Moreover, the possibility for attackers to exploit and take control of the system is high due to self-configuration and automation. Furthermore, the storage of industrial production-related data on the cloud is a problem for data privacy [200]. As a result, industrial internet software must secure linked devices and generate data against various threats. Furthermore, to secure automation processes continually, security upgrades must not interfere with control processes and must be seamlessly integrated with the usual control cycle.

## 8. Conclusions

IoT technology is expanding at a fast-paced rate and various testbeds have been developed to improve smart industry productivity. With a large number of studies published over recent years, a comprehensive overview of the state-of-the-art technologies for IIoT in the industry holds significant importance. This study examined state-of-the-art IIoT network architecture, platforms, topologies, and protocols that enable the smart industry to improve manufacturing productivity by facilitating access to the IoT backbone. Furthermore, we include a comprehensive review of the present and future developments in industrial IoT applications, devices/sensors, communication protocols, and many other linked technologies. Consequently, for a better understanding of IoT smart industry security, we covered a variety of industrial IoT challenges and security requirements.

The analysis reveals several important and crucial aspects of IoT-based industries and key technologies, such as cloud computing, big data storage, and analytics. It is found that energy consumption, data privacy and confidentiality, network latency, and high connectivity are major challenges in IIoT. With the expanding network, scalability is an obvious challenge for the IIoT network. Moreover, due to the heterogeneity of sensors deployed in the IIoT network, interoperability is becoming challenging. Due to the shorter span of consumer IoT devices, implementing complex security protocols is also difficult and raises security and privacy issues.

Recently, governments have begun to support IIoT, and in the near future, traditional industrial techniques are anticipated to be transitioned into the IoT industry. Moreover, many popular firms also began investing and creating new strategies to improve manufacturing productivity using IoT technologies. Finally, researchers, experts, industrialists, and policymakers working in the IoT sector and industrial technologies are likely to find this comprehensive survey to be highly important and helpful.

This survey provides a comprehensive overview of the role of IoT in the manufacturing industry in general. Key components of IIoT smart industry are discussed concerning their features and flaws. Existing surveys explore different aspects of IIoT, like security challenges, blockchain-based solutions, software-based and fog-based IIoT solutions, etc.; this survey provides an extensive survey of attacks, weaknesses, and vulnerabilities of IIoT and provides probable solutions to overcome these issues. With an increased number of articles publishing rapidly, the survey might have missed the most recent articles on IIoT security. The survey covered only traditional solutions for IIoT attacks. Quantum computing-based attacks have been launched recently and traditional security protocols are unable to detect such attacks. Exploring quantum cryptography solutions to overcome such attacks on IIoT would be an interesting avenue.

## Figures and Tables

**Table 1:** Comparative analysis of existing related work.

Ref.	IIoT Security	Major Attacks	Countermeasures	Blockchain	Software-Based IIoT	Fog-Based IIoT
[10]	Yes	No	Yes	No	No	No
[11]	Yes	No	Yes	No	No	No
[12]	Yes	Yes	Yes	No	No	No
[13]	No	No	Yes	No	Yes	Yes
[14]	No	No	Yes	No	Yes	Yes
[15]	No	Yes	Yes	No	No	Yes
[16]	Yes	No	No	Yes	No	No
[17]	Yes	No	No	Yes	No	No
Current	Yes	Yes	Yes	Yes	Yes	Yes

**Table 2:** Comparison between current wireless protocols.

Protocols	Frequency Band	Standards	Transmission Range	Data Rate	Cost	Energy Usage
ZigBee	2.4 GHz	IEEE 802.15.4	10–20 m	20–250 Kilobyte	Low	Low
Bluetooth	24 GHz	IEEE 802.15.1	8–10 m	1–24 Mbs	Low	Very Low
WIFI	5–60 GHz	IEEE 802.11	20–100 m	1 Mbegabite–7 Gigabyte	High	High
MQTT	2.4 GHz	OASIS	-	250 kilobyte per second	Low	Low
Lora WAN	868/900 MHz	Lora WAN R1.0	<30 KM	0.3–50 Kb per second	High	Very Low
Mobile Cellular Networks	865–MHz, 2.4 GHz	2G–GSM, CDMS–3GUMTS, CDMA2000, 4G–LTE	Entire Cellular Area	2G: 50–100 Kb per second 3G 200 Kb per second 4G: 0.1–1 Gb/s	Medium	Medium
RFID	860–960 MHz	ISO 18,000–6C	1–5 m	40–160 Kb per second	Low	Low
WiMAX	2 GHz–66 GHz	IEEE 802.16	<50 KM	1 Mb per second–1 Gb per second (Fixed) 50–100 Mb/s (mobile)	High	Medium
LR–WPAN	868/915–MHz, 2.4 GHz	IEEE 802.15.4	10–20 m	40–250 Kb per second	Low	Low

**Table 3:** Industrial IoT sensor-based applications description.

Sensors	Description of Sensors, IoT Connections/Roles
Temperature Sensor	One of the most important requirements for this sensor is to help prevent moisture on a large production floor. In addition, temperature sensors also help detect extremely high temperatures in manufacturing processes and display our performance rating [97].
Humidity Sensor	Humidity sensors, which monitor the quantity of moisture in the air, are the most useful IIoT sensors. The humidity would build in our customer’s application, and the flooring would become completely soaked. The production line was severely affected by wet feet. IIoT humidity sensors could be used during the production line to monitor the humidity [98].
Pressure Sensor	IIoT sensors generally require the ability to read pressure. Therefore, choosing the correct pressure sensor for every application, from detecting air pressure to harmful gases and liquids, requires some research. In industrial applications, pressure sensor designs detect leaks or flow blockages. Other transmissions may be issued if pressure fluctuations surpass predefined limitations. Pressure sensors provide a fast payback period, especially when faults are found [99].
Current- monitoringSensor	When IIoT sensor procedures are used, power consumption monitoring cannot be minimized [100]. The current monitoring method helps you to check utility bills. Unfortunately, the current monitoring devices do not help predict the system’s failure [101]. When an application fails on an industrial motor, the first thing that happens is friction. A larger load on an engine is caused by friction. When power consumption exceeds expected levels, motor utilization can detect failures. The most significant utilization evidence has come from industrial freezers. When compressors fail, for example, one of two things can happen: current consumption is significantly lowered (allowing the motor to spin freely without load) due to internal component failure, or recent consumption increases due to friction [102].
VibrationSensor	Vibration sensors are crucial components of IIoT sensors. Vibration sensors can alert the user to frequent faults with working machinery and devices, making them a solution for many predictive preservation applications. Accelerometers are used in vibration sensors to read microchanges over a wide range of frequencies. NCD vibration sensors can detect malfunctioning items from heavy machinery and motors to industrial pipe flow vibration monitoring. However, this sensor can save lives when utilized appropriately, making it the top-ranked sensor for predictive maintenance applications because of its early detection abilities. Furthermore, the vibration sensor is the most commonly utilized in industrial applications that do not require human intervention [103].
Water-DetectionSensor	Water-detection sensors are essential sensors for industrial applications. When water is exposed, they send an alert, and when the sensor has been restored to its dry state, they send another alert. Water detection sensors also communicate data regularly, letting you know they are still watching out for you. The battery state is also communicated, as it is with all NCD sensors, to control the sensor’s overall health. Water detection sensors have been used to detect floods in unexpectedly large numbers of applications. In addition, this sensor is commonly used to detect water in basements. Detecting water on solid floors and walls is one of the most fundamental detecting applications [104].

**Table 4:** Countermeasures of physical layer attacks.

Ref.	Countermeasures/Solutions	Physical Attacks	Effects
[121]	NOS middleware	Permanent denial-of-service (PDoS)	Resource destruction
[122,123]	Support vector machine (SVM)	Sleep denial	Node shutdown
[124]	CUTE Mote; packets’ rerouting to alternative routes	RF interference/jamming	DoS; hinder/jam communication
[125]	Masking technique; authentication using PUF	Side-channel attack	Collect encryption keys
[127]	PAuthKey	Fake node injection	Control data flow; man-in-the-middle
[128]	PUF-based authentication	Malicious code injection and physical tampering	DoS attacks; leak sensitive information

**Table 5:** Countermeasures of network layer attacks.

Ref.	Countermeasures/Solutions	Network Attacks	Effects
[139]	Privacy-preserving traffic obfuscation framework	Traffic analysis attack	Data leakage
[140]	SRAM-based PUF	RFID spoofing and unauthorized access	Data manipulation and modification (read, write, delete)
[141]	Hash chain authentication	Routing information attacks	Routing loops
[142]	Hash chain authentication; monitor-based approach	Selective forwarding	Message destruction
[143]	Hash chain authentication; intrusion detection	Sinkhole attack	Data alteration or leakage
[144]	Clustering-based intrusion detection system	Wormhole attack	Packet tunneling
[145]	Trust aware protocol	Sybil attack	Unfair resource allocation; redundancy
[146,147]	Secure MQTT; inter-device authentication	Man-in-the-middle attack	Data privacy violation
[148]	Signcryption	Replay attack	Network congestion; DoS
[149]	DDoS server; SDN-based IoT framework	DoS/DDoS attack	Network flooding; network crash

**Table 6:** Countermeasures of software and data link layer attacks.

Refs.	Countermeasures/Solutions	Physical Attacks	Effects
[154,155]	Lightweight framework; high-level synthesis (HLS)	Trojan horses, virus, adware, worms, and spyware.	Resource destruction
[156,157]	Lightweight neural network framework; malware image classification	Malware	Infected data
[158]	Privacy-preserving ABE; blockchain-based ABE	Unauthorized access	Violation of data privacy
[159,160]	Two-factor authentication; DPP; ISDD	Data breach	Data leakage
[161,162]	Chaos-based scheme; blockchain architecture	Data inconsistency	Data inconsistency

**Table 7:** Description of IIoT trends and directions in some popular technology industries.

Firms	Directions and Trends
IBM, Armonk, NY, USA	IBM can increase process product quality, capabilities, and insights, decrease production errors, and save money and time by applying AI-powered visual inspection of components and assemblies. Quality control workers can use a smartphone connected to the cloud to monitor manufacturing operations from anywhere at no cost. Furthermore, manufacturers can spot mistakes earlier rather than later using machine learning algorithms when more expensive repair work is needed [171].
Intel, Santa Clara, CA, USA	Intel can help quicken the time of value data-driven, interoperable IIoT solutions. The ecosystem of innovators and a collection of flexible solutions help develop and integrate intelligent industrial edge solutions that reduce costs, increase profits, and move you ahead of the competition. In addition, Intel enables the deployment of smart factory solutions to achieve new productivity levels while exposing new opportunities to maximize income [172].
Samsung, Seoul, Korea	Samsung takes action in the world of IoT. Samsung SDS’s IoT platform lets users connect with various devices and many IoT communication protocols like Zigbee, Lora WAN, MQTT, BLE, and Modbus [173].
Oracle, Austin, TX, USA	Oracle’s digital world applications include customer experience (CX), supply chain, HR, and ERP to increase operational efficiency, boost worker productivity, improve CX, generate new business models and prospects, and support intelligent, predictive algorithms, and digital twins [174].
Microsoft, Redmond, WA, USA	Microsoft is the reason behind the digital transformation of smart manufacturing to improve in productivity and grow industrial processes. In addition, Microsoft also helps IIoT sensors communicate with artificial intelligence (AI) to create smart machines and equipment that communicate. In addition, since IIoT generates massive volumes of big data, it needs a fast, powerful system [175].
HQ Software, New York, NY, USA	HQ Software gives solutions for IIoT services to make the whole process of manufacturing more efficient. One of the efficiency parameters is a shorter manufacturing cycle; IIoT results in choosing the right IoT automation software to decrease the manufacturing cycle time and cut costs [176].
Cisco, San Jose, CA, USA	Cisco gives a solution for a secure and strong network infrastructure for the success of Industry IoT [177].
Google, Mountain View, CA, USA	Google Cloud Open System infrastructure provides an IIoT solution for developing opportunities, new devices, technologies, and business models.

## Data Availability

Not applicable.
